# Hypovigilance Detection for UCAV Operators Based on a Hidden Markov Model

**DOI:** 10.1155/2014/567645

**Published:** 2014-05-20

**Authors:** Yerim Choi, Namyeon Kwon, Sungjun Lee, Yongwook Shin, Chuh Yeop Ryo, Jonghun Park, Dongmin Shin

**Affiliations:** ^1^Department of Industrial Engineering, Seoul National University, Seoul 151-744, Republic of Korea; ^2^Samsung Electronics, Suwon, Gyeonggi-do 443-742, Republic of Korea; ^3^Agency for Defense Development, Daejeon 305-152, Republic of Korea; ^4^Department of Industrial and Management Engineering, Hanyang University, Ansan, Gyeonggi-do 425-791, Republic of Korea

## Abstract

With the advance of military technology, the number of unmanned combat aerial vehicles (UCAVs) has rapidly increased. However, it has been reported that the accident rate of UCAVs is much higher than that of manned combat aerial vehicles. One of the main reasons for the high accident rate of UCAVs is the hypovigilance problem which refers to the decrease in vigilance levels of UCAV operators while maneuvering. In this paper, we propose hypovigilance detection models for UCAV operators based on EEG signal to minimize the number of occurrences of hypovigilance. To enable detection, we have applied hidden Markov models (HMMs), two of which are used to indicate the operators' dual states, normal vigilance and hypovigilance, and, for each operator, the HMMs are trained as a detection model. To evaluate the efficacy and effectiveness of the proposed models, we conducted two experiments on the real-world data obtained by using EEG-signal acquisition devices, and they yielded satisfactory results. By utilizing the proposed detection models, the problem of hypovigilance of UCAV operators and the problem of high accident rate of UCAVs can be addressed.

## 1. Introduction

Over the last decade, the advances in military technology have brought widespread proliferation of unmanned combat aerial vehicles (UCAVs) which can deliver weapons or attack targets without on-board operators [1, 2]. They have not only enabled an operator to control aerial vehicle (s) in a remote manner, contributing to the decrease of human casualties, but also expanded operational ranges of surveillance and reconnaissance [3]. UCAVs, however, have shown a high accident rate which is 10 to 100 times higher than that of manned aerial vehicles [4], causing the operation costs to be more than double compared to manned aerial vehicles [5]. It is also known that, due to the complex and diverse tasks of UCAVs, they show much higher accident rate than unmanned aerial vehicles [6]. Therefore, the high accident rate becomes a major concern for both UCAV operators and their administrators.

To address the problem of the high accident rate, several studies are conducted to investigate the causes of such high accident rate [7, 8]. These studies suggest that almost 20% of accidents related to UCAVs are due to erroneous decision-making resulting from human factors such as lack of situation awareness and heavy workload [9]. In particular, one of the main reasons for the high accident rate is reported to be the occurrence of the hypovigilance phenomenon [10].

Vigilance refers to a mental state where an operator maintains attention while performing tasks over prolonged periods of time [11] and vigilance level changes continuously during the course of the operation [12]. As such, vigilance level of an operator can become lower over time, and this phenomenon is known as the hypovigilance. Furthermore, vigilance level of an operator is known to get lowered even when the operator is highly motivated [13]. Therefore, careful consideration needs to be taken in order to prevent hypovigilance by maintaining the operator's vigilance at a desirable level. In case of traffic accidents, it has been reported that about 25% of road crashes are caused by hypovigilance of car drivers [14]. Also, hypovigilance can cause a serious decrease in task performance of operators [15].

As aforementioned, hypovigilance is one of the major causes of accidents in the operation of UCAVs resulting in high cost of operations; it is therefore critical for UCAV operators who suffer from lower tension levels due to increasing fatigue to sustain an appropriate vigilance level [10]. For these reasons, detecting hypovigilance of UCAV operators is not only an important topic for research but also one of the key factors in operating UCAVs. Three types of operators are usually involved in UCAV operations which include a pilot, a mission specialist, and a flight director [16]. Among them, in this paper, hypovigilance of a UCAV pilot who maneuvers the vehicle him/herself is considered.

Two important characteristics should be considered in developing hypovigilance detection models for UCAV operators. First, the process of detection should be performed with extremely short latency. For effective recovery of the operator's vigilance level, hypovigilance of the operator needs to be detected at the time of occurrence or beforehand. Second, the detection should be conducted without intruding on the operator's tasks in any way. Since maneuvering UCAVs usually involves several simultaneous complicated tasks, additional burden brought by vigilance detection can have a negative impact on the operator's performance.

To investigate an operator's vigilance level, we use electroencephalography (EEG) signal. EEG signal which refers to the recordings of electrical activity along the scalp is a widely used data related to human mental states such as vigilance [17, 18]. EEG signal is utilized in this paper for the following reasons. First, the signal can be obtained in a real-time manner [19], making it possible to detect hypovigilance with short latency. Second, the signal can be acquired without intrusion as there are several EEG-signal acquisition devices which take the form of a headset [20]. Third, compared to other vigilance detection measures, EEG signal contains minimal bias [21] since it directly shows human brain activities. Therefore, by using EEG signal, the hypovigilance of an operator can be detected with short latency, minimal impact on his/her UCAV maneuvering tasks, and minimal bias.

**Figure 1:**
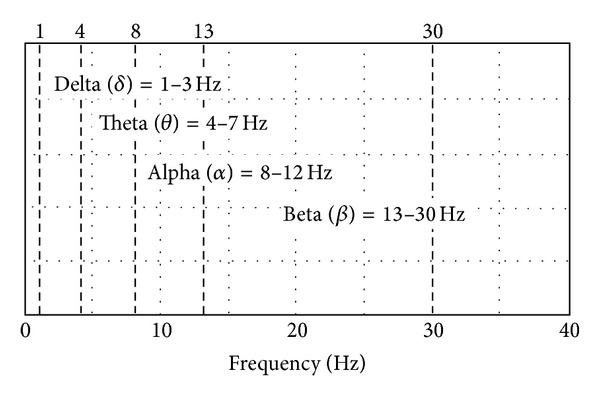
Frequency ranges of the four bands, delta (0–3 Hz), theta (4–7 Hz), alpha (8–12 Hz), and beta (13–30 Hz), of EEG signal.

**Figure 2:**
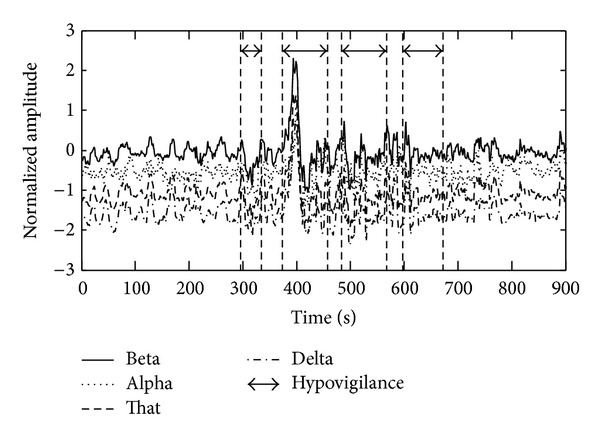
Example of EEG signal for an operator, where sequences enclosed by arrows indicate hypovigilance states of the operator.

Moreover, EEG signal is composed of four frequency bands, delta (0–3 Hz), theta (4–7 Hz), alpha (8–12 Hz), and beta (13–30 Hz), as shown in Figure 1. They were acquired after filtering raw EEG signal by a bandpass filter with bandwidth ranging from 0 to 30 Hz and performing wavelet packet decomposition [22]. An example of EEG signal of an operator is shown in Figure 2, where the four lines, respectively, indicate normalized amplitudes of the four frequency bands, and the sequences enclosed by arrows are signals generated from an operator in case of hypovigilance. Among constantly changing amplitudes of EEG signal, the sequences of EEG signal enclosed by the four arrows show particularly drastic differences between maximum and minimum amplitudes. As such, learning the pattern of EEG signals according to vigilance levels by using statistical learning method is expected to be effective. Note that hypovigilance states in the graph were marked manually by the subject involved in the experiment for illustrative purpose since there is no unanimously agreed measure of vigilance levels.

Nevertheless, detecting hypovigilance of UCAV operators based on EEG signal is challenging because of the following reasons. First, EEG signal is sequential and nonstationary data, meaning that it varies depending on when it is measured [23], and the signal patterns change rapidly over time [24, 25]. Second, it is almost impossible to obtain labels which indicate vigilance levels for every interval of EEG signal since constant intervention should be avoided. Last but not least, EEG signal is specific to an individual, meaning that it generates different patterns depending on operators [26]. For the last reason, a hypovigilance detection model which is appropriate for one operator cannot be applied to other operators.

In this paper, we exploit hidden Markov models (HMMs) to develop hypovigilance detection models for UCAV operators using EEG signal. HMMs which utilize probabilistic models of sequential data have been used in a variety of machine learning applications. Several studies show that HMMs are appropriate for modeling EEG signal due to their ability to deal with dynamics in observation sequences [27, 28].

Particularly, to alleviate the problem of unavailability of labels for EEG signal, we adopt the hypothesis that higher vigilance level is required to perform more difficult tasks as suggested by Galin et al. [29]. Specifically, the vigilance levels of EEG signal generated while performing relatively difficult tasks and easy tasks are labeled as normal vigilance and hypovigilance, respectively. The clear difference among the difficulty levels of distinct UCAV tasks compared to that of UAVs due to the diversity of the tasks can contribute to the accurate labeling of hypovigilance, resulting in better detection performance.

In addition, two HMMs are then trained to represent each of the two levels. Then, the detection model based on the two trained HMMs is used to detect hypovigilance of an operator. This process is performed for each individual operator in order to consider the different patterns of EEG signal for each operator.

This paper is organized as follows. In Section 2, studies related to hypovigilance detection methods and EEG-signal classification methods are presented. Then, HMM-based detection models for hypovigilance of UCAV operators are proposed in Section 3 along with a brief introduction of HMMs and their fundamental algorithms. In Section 4, two sets of experiments are conducted, one experiment for validation of the vigilance level hypothesis and the other for performance evaluation of the proposed models. Lastly, this paper is concluded in Section 5.

## 2. Literature Review

### 2.1. Hypovigilance Detection Methods

To the best of the authors' knowledge, there is limited direct research on hypovigilance detection of UCAV operators. However, several research efforts have been made for the detection of hypovigilance in other areas such as car driving [30–32] and game playing [33]. These studies can be classified into four categories depending on the data used for the detection, survey data, physical data, performance data, and biological data as shown in Table 1.

Survey data is usually collected during or after a given operation through self-assessment by the operators using predefined scales for vigilance levels such as the Karolinska sleepiness scale [34]. In spite of the convenience in collecting survey data, it has a drawback as pointed out in Craig et al. [35] that psychological factors can affect the self-assessed data, resulting in discrepancy between the actual vigilance level and the self-assessed vigilance level. We note that obtaining survey data in the process of maneuvering UCAVs is intrusive to the operator's tasks and nearly impossible to be done in real time as well.

To mitigate the problem of studies using survey data, several efforts have been made using physical data or task performance data. These methods usually intend to exploit observed data with the assumption that the vigilance level of an operator induces changes in physical features or task performance. Various physical data including duration, amplitude, and velocity of eye blinking [30, 36] and performance data such as road center position and vehicle velocity [31, 37] are used for the detection of hypovigilance. Even though these types of data can be acquired in real time in a less intrusive manner, there still exists an inherent bias between the actual vigilance level and the conjectured vigilance level from physical or performance data [38] due to weather conditions or level of difficulty of the tasks.

Biological data such as EEG, electrocardiogram (ECG), and electrooculography (EOG) are also used as indicators of hypovigilance in several studies [39–41]. Although biological data is sensitive to stimulus from environment, causing noises [42], it can be obtained in a less intrusive manner possibly in real time. Moreover, since the biological data can be used as an external indicator of human brain activities, bias between actual and conjectured vigilance levels tends to be small.

Among the biological data, EEG signal is widely accepted as an effective and efficient indicator of the transition between wakefulness and sleep as well as between the different sleep stages [43]. Furthermore, EEG signal shows different patterns depending on various actions or mental states, and it is used for monitoring dynamic fluctuations in cognitive states including vigilance level and mental workload [39, 44].

### 2.2. EEG-Signal Classification Methods

Diverse classifiers have been used in EEG-signal classification, and these classifiers can be divided into two types, static classifiers and dynamic classifiers. Static classifiers categorize an instance based on a single feature vector, while dynamic classifiers find the optimal category for an instance by considering a sequence of feature vectors over a time period [24]. Moreover, the dynamic classifiers are known to be more appropriate in modeling sequential information [45].  Table 2 shows previous studies for classifying EEG signal in terms of their classifier type, methods, and applications.

For static classifiers with EEG signal, support vector machines and neural networks are applied to classify the state of emotion into several groups [46, 47], and naive Bayes and Fisher discriminant analysis are also used to detect arousal of emotion [48]. In Yeo et al. [44], support vector machines are used to detect the driver's drowsiness during car driving, and Faust et al. [17] applied support vector machines, Gaussian mixture models, and neural networks for the detection of epileptic activity by using EEG signal.

On the other hand, various dynamic classifiers have been employed in the domains where sequential information is important for EEG-signal classification. Barreto et al. [49] present time-delay neural networks and gamma neural networks for classifying changes in EEG signal that is generated by voluntary movement. HMMs are applied to detect the changes in the vehicle driver's state of arousal [50], and variations of HMMs called input-output HMMs are also used to distinguish EEG-signal changes between three cognitive and motor-related mental tasks [51]. They consider a situation where the distribution of the output variables and the states are dependent on input variables.

Several studies which compare static and dynamic classifiers have suggested that the dynamic classifiers show more promising results than the static classifiers when noises are found in the data. Obermaier et al. [52] present HMMs for online classification of single trial EEG signal generated by intension for left- or right-hand movement. They show that a HMM-based model outperforms linear discriminant analysis-based model in terms of error rates through experiments. In addition, Št'astný et al. [53] classify EEG signal into two types of movements, the distal and the proximal movements, using HMMs and neural networks and demonstrate that the performances of HMMs are better than those of neural networks.

As such, HMMs have been widely used for classifying EEG signal. Doroshenkov et al. [54] conduct EEG-signal classification of human sleep stages based on HMMs and show reliable identification accuracy of the main stages of sleep. Furthermore, Lederman and Tabrikian [55] demonstrate that HMMs are suitable for detecting nonstationary changes of EEG signal and suggest that HMMs are one of the best methods for time series classification.

## 3. Hypovigilance Detection Models for UCAV Operators

### 3.1. Problem Definition

In this section, we present the hypovigilance detection problem of UCAV operators by introducing formal notations for operators, tasks, and sequences of EEG signal.

We denote a set of operators by *O* = {*o*_*m*_ | *m* = 1,…, *M*} where *M* indicates the number of operators considered in the problem. Each operator performs a set of tasks, denoted by *T* = {*t*_*n*_ | *n* = 1,…, *N*}, where *N* represents the number of tasks, and a task *t*_*n*_ is composed of a set of steps *σ*_*nk*_ for *k* = 1,…, *K*, where *K* indicates the number of steps in a task. Each task has a predefined difficulty level which takes one of two possible values, easy or difficult. As such, a sequence of the EEG signal which is generated from the *m*th operator, *o*_*m*_, who has completed the *n*th task, *t*_*n*_, is denoted by *E*_*mn*_, which is composed of **e**_*mnl*_, a vector of EEG-signal values from multiple channels at time *l* such that *l* = 1,…, *L*.

According to the hypothesis on the relation between the difficulty level of a task and the corresponding vigilance level, the state of an EEG-signal sequence generated while performing difficult or easy tasks is assumed to be normal vigilance or hypovigilance, respectively. The vigilance level of an EEG-signal sequence, *E*_*mn*_, is denoted by *ν*_*mn*_ ∈ {+, −} where plus sign indicates normal vigilance and minus sign indicates hypovigilance. Therefore, a set of EEG-signal sequences obtained from *o*_*m*_, denoted by **E**_*m*_ = {*E*_*mn*_ | *n* = 1,…, *N*}, is divided into two sets, one of normal vigilance EEG-signal sequences, **E**_*m*_^+^, and the other of hypovigilance EEG-signal sequences, **E**_*m*_^−^.

In this paper, the HMM-based hypovigilance detection problem for UCAV operators refers to developing models which classify the vigilance levels of EEG-signal sequences into one of the two states, normal vigilance or hypovigilance. Specifically, given a sequence of EEG signal from *o*_*m*_ performing *t*_*n*′_, *E*_*mn*′_ = **e**_*mn*′1_ **e**_*mn*′2_,…, **e**_*mn*′*L*_, the hypovigilance detection model determines the vigilance level of the sequence, *ν*_*mn*′_, by means of trained HMMs which have been constructed using **E**_*m*_^+^ and **E**_*m*_^−^.

### 3.2. Continuous HMM-Based Hypovigilance Detection

For the hypovigilance detection models of UCAV operators, we adopt HMMs which are widely used statistical learning based classification methods [45]. They are statistical Markov models which assume the Markov property, where the next state is determined only by the current one, not by previous states, and they have been successfully applied to various sequential data processing problems such as speech recognition [56] and signal processing [57].

In this paper, by considering that an EEG-signal sequence is a sequence of continuous values, continuous HMMs are employed for the detection. A continuous HMM is denoted by Λ = (*S*, *X*, *A*, *B*, Π) where *S*, *X*, *A*, *B*, and Π represent a set of hidden states, a set of observation values, a state transition probability matrix, an observation probability density matrix, and an initial state probability vector, respectively.

Specifically, *A* = [*a*_*ij*_] is a matrix of state transition probabilities whose element *a*_*ij*_ represents the probability of transition from state *s*_*i*_ to state *s*_*j*_ for *i*, *j* = 1,…, *G*, and it is calculated as

(1) $$\begin{array}{c} a_{ij}=P\left(q_{l+1}=s_{j}\;|\;q_{l}=s_{i}\right), \end{array}$$

where *q*_*l*_, *l* = 1,…, *L* − 1, is a state at time *l* and *G* is the number of possible states.

Moreover, *B* = [*b*_*j*_(**x**)] is a matrix of observation probability densities, and each element *b*_*j*_(**x**) represents the probability density of emitting observation vector **x** in state *s*_*j*_ for **x** ∈ **R** ^*k*^ where *k* indicates the dimension of **x** and *j* = 1,…, *G*. It is represented by the following equation:

(2) $$\begin{array}{c} b_{j}\left(x\right)=f\left(e_{l}=x\;|\;q_{l}=s_{j}\right), \end{array}$$

where *e*_*l*_, *l* = 1,…, *L*, is an observation made at time *l*.

Π = [*π*_*i*_] is a vector of initial state probabilities, whose element *π*_*i*_, *i* = 1,…, *G*, is calculated by the following equation:

(3) $$\begin{array}{c} \pi_{i}=P\left(q_{l}=s_{i}\right), \end{array}$$

which indicates the probability that the initial state of a sequence is *s*_*i*_.

**Figure 3:**
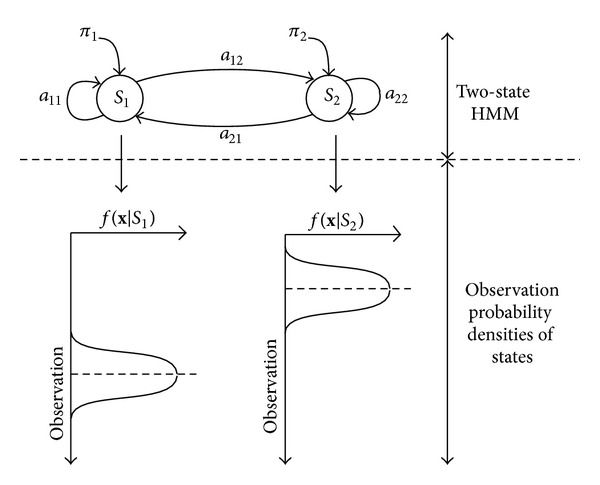
Example of continuous HMM with two states.

Figure 3 shows an example of continuous HMMs with two states, *s*_1_ and *s*_2_. From the continuous HMM, Λ = (*S*, *X*, *A*, *B*, Π), two different kinds of sequences are generated. The first one is a state sequence denoted by *Q* = *q*_1_ *q*_2_,…, *q*_*L*_, which is composed of unobservable elements from a set of hidden states *S*. The second one is an observation sequence denoted by *E* = **e**_1_ **e**_2_,…, **e**_*L*_, which is generated from aforementioned state sequence according to the observation probability density of each state.

For the detection of hypovigilance from EEG signal by using HMMs, two approaches can be applied. First, state-based approach regards states as vigilance levels so that single or multiple states indicate a single level. Second, the model-based approach associates an HMM with a specific vigilance level. The latter approach has been used in a variety of domains including EEG-signal classification for mental fatigue [40] and trend analysis of technologies [58]. In this paper, both approaches are adopted in such a way that the state-based approach is used to validate the vigilance level hypothesis and the model-based approach is used for constructing hypovigilance detection models.

In the state-based approach, an HMM, Λ = (*S*, *X*, *A*, *B*, Π), is trained with a set of observation sequences, **E**, regardless of their classes to estimate the parameters of the HMM, *A*, *B*, and Π, such that the most likely sequence of hidden states, *Q*, of a given observation sequence, *E*, can be identified. To this end, various algorithms in the frame of expectation-maximization algorithm [59] are exploited such as Baum-Welch algorithm [60] and *k*-means algorithm [61]. After an HMM is trained, Viterbi algorithm [62] is used to find the most likely *Q* of the given *E* from the trained Λ.

In the model-based approach which considers the same number of HMMs as that of vigilance levels, an observation sequence is classified as a level represented by the HMM which has the highest likelihood for that sequence. Observation sequences of the same level are used to train the corresponding HMM by means of the same training algorithms used in the state-based approach. We note that the likelihood of an observation sequence, *E*, with a trained HMM, Λ, *f*(*E* | Λ), is determined by using the forward or backward procedures [45].

### 3.3. Hypovigilance Detection Models

In this section, we describe the HMM-based hypovigilance detection models constructed in this paper. As aforementioned, hypovigilance which refers to a mental state of an operator below a desired vigilance level tends to occur in the process of performing relatively undemanding tasks.

Observation values in a sequence of EEG signal are assumed to be mutually independent and to follow the Gaussian distribution. The Gaussian distribution assumption has been widely used for modeling of EEG signal in several studies [24, 63]. Particularly, since the observation values in this work are a vector composed of continuous values generated from EEG channels, the observation probability densities are assumed to follow a multivariate Gaussian distribution with known mean, *μ*_*j*_, and variance, Σ_*j*_, *j* = 1,…, *G*, where *G* indicates the number of possible states.

**Figure 4:**
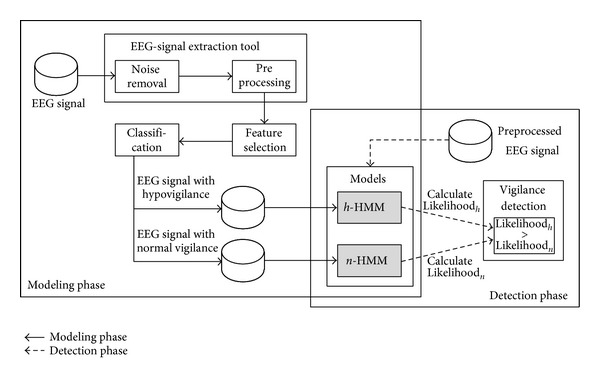
Overview of the HMM-based hypovigilance detection models including model training phase and model testing phase for two HMMs, *h*-HMM, and *n*-HMM.

Considering that EEG-signal patterns vary depending on individuals [26], we construct a specific detection model for each UCAV operator. In detail, EEG-signal sequences acquired from an operator are considered as observation sequences representing his/her mental state and only these are used to train the operator's detection model. Moreover, we utilize two HMMs for a detection model of an operator, corresponding to two different vigilance levels.  Figure 4 shows the overview of how the two HMMs (e.g., *n*-HMM and *h*-HMM) play a role in detecting hypovigilance of an operator by using EEG-signal sequences obtained from the operator. The process of hypovigilance detection can be divided into two phases, the modeling phase and the detection phase.

In the modeling phase, two HMMs are trained by using EEG-signal sequences to estimate the parameters of each HMM. Specifically, EEG signal is collected from an operator, *o*_*m*_, and preprocessed from EEG-signal extraction tools. Appropriate features are then selected, which are sequences of EEG-signal, *e*_*mnl*_, for all *l* = 1,…, *L*, generated while the operator performs tasks, *t*_*m*_, for all *m* = 1,…, *M*. The two HMMs, *n*-HMM and *h*-HMM, for *o*_*m*_ are trained by using respective EEG-signal sequence sets, **E**_*m*_^+^ and **E**_*m*_^−^, which are categorized in advance. The *n*-HMM is denoted by Λ_*m*_^+^ = (*S*_*m*_^+^, *X*_*m*_^+^, *A*_*m*_^+^, *B*_*m*_^+^, Π_*m*_^+^), while *h*-HMM is denoted by Λ_*m*_^−^ = (*S*_*m*_^−^, *X*_*m*_^−^, *A*_*m*_^−^, *B*_*m*_^−^, Π_*m*_^−^). Their parameters are estimated by means of *k*-means algorithm [61] whose initial parameters are set to random values between maximum and minimum observation values.

In the detection phase, the likelihood of a given sequence, *E*_*mn*′_, for two trained HMMs is calculated, and the vigilance level of *E*_*mn*′_ is determined by comparing the likelihood of the two. The likelihoods, *Likelihood*_*h*_ and *Likelihood*_*n*_, are defined as *f*(*E*_*mn*′_ | Λ_*m*_^−^) and *f*(*E*_*mn*′_ | Λ_*m*_^+^), respectively. As such, the vigilance level of *E*_*mn*′_ is determined by the following equation:

(4) $$\begin{array}{c} \text{Class}\left(E_{mn^{\prime }}\right)={\text{argmax}}_{\nu \in \{-,+\}}f\left(E_{mn^{\prime }}\;|\;\Lambda_{m}^{\nu }\right), \end{array}$$

where Class(*E*_*mn*′_) is a function that returns the vigilance level of *E*_*mn*′_.

Aforementioned procedures of vigilance level classification are shown in Algorithm 1.

## 4. Experiments

In this section, we present two experiments to illustrate the efficacy and efficiency of the HMM-based hypovigilance detection models. The first phase of the experiment intends to validate the hypothesis on the vigilance level, which suggests that the vigilance level of an operator becomes higher as the operator encounters more difficult tasks, while it becomes lower when the operator encounters relatively easy tasks. Second, accuracies of the proposed detection models are assessed by using EEG signal acquired from more complex settings to evaluate the performances of the detection models.

### 4.1. Adoptability Validation

To validate the adoption of the vigilance level hypothesis suggested in [29] to our problem, trends in the vigilance levels of the operators with respect to the difficulty levels of tasks are observed by computing state transition probabilities from the state-based classification of EEG-signal sequences obtained from operators.

#### 4.1.1. Dataset

**Figure 5:**
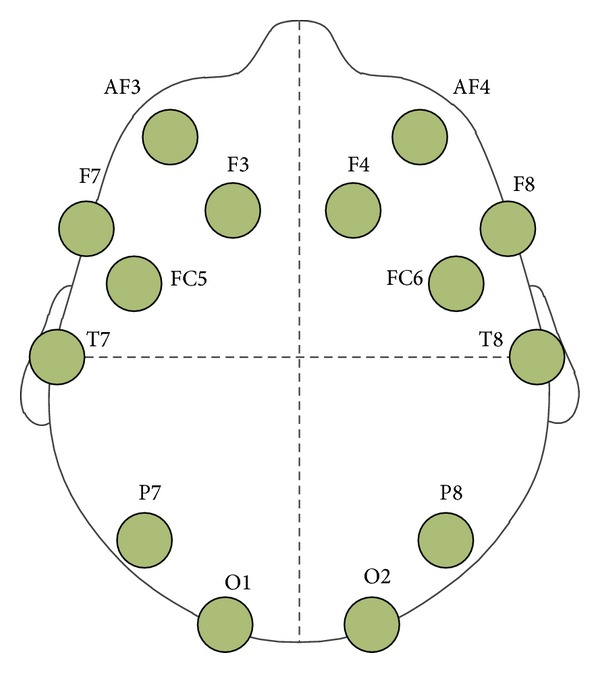
Diagram showing the positions of the 14 channels, where EEG signal was recorded by EEG-signal acquisition device, Emotiv EPOC.

EEG signal was recorded from two subjects who are both right-handed males, aged twenty-four and twenty-eight, respectively, by using EEG-signal acquisition device, Emotiv EPOC [20]. Emotiv EPOC is a nonintrusive brain computer interface tool, which extracts EEG signal from 14 channels located in different positions of the scalp, O1, O2, P7, P8, T7, T8, FC5, FC6, F7, F8, F3, F4, AF3, and AF4, according to the international 10–20 system as shown in Figure 5. Each EEG-signal sequence used in this paper is a vector composed of power spectrum values generated from the 14 channels aforementioned. The signals during the first and the last 5 seconds of the experiments and the signals determined to contain noises from visual inspections were truncated. In the ratio of 2 : 1, the signals were randomly divided into training and test datasets.

The two subjects performed four types of tasks with different levels of difficulty, which include cruising, taking off, landing, and emergency landing according to the level of difficulty from low to high. The tasks are provided by a flight simulator called Falcon 4.0 [27] and are designed to be similar to those of real UCAVs. The order of the level of difficulty of the different tasks was judged by an expert UCAV operator.

#### 4.1.2. Vigilance Level Trends

In this experiment, to find the trends between the vigilance level of an operator and the level of difficulty of a given task, the state probability of each state is obtained, where the state probability indicates the probability of being in a certain state in a HMM. States 1 and 2 are associated with the cases of normal vigilance and hypovigilance, respectively.

**Figure 6:**
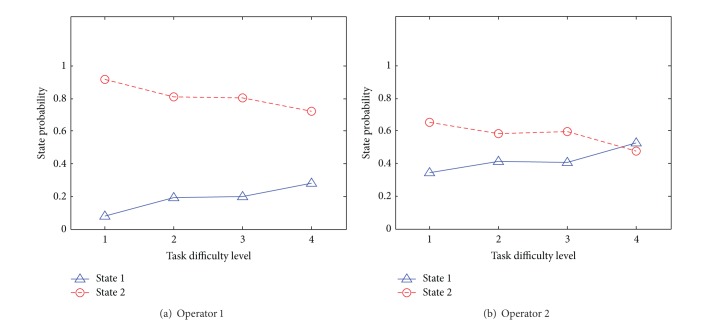
Experiment results showing the trends in state probabilities of (a) operator 1 and (b) operator 2 while performing tasks with various difficulty levels, where 1 indicates the least difficult task and 4 indicates the most difficult one.

Changes in state probabilities of states 1 and 2 according to the difficulty level of the tasks for two subjects, operator 1 and operator 2, are illustrated in Figure 6. The numbers in the horizontal axis indicate the difficulty level of tasks, where 1 means the least difficult task, while 4 means the most difficult one. From the experiment results, it can be observed that operator 1 has maintained the same vigilance level regardless of the difficulty level of the tasks as the state probability of state 2 ranges between 0.8 and 1.0 for all difficulty levels, which are always above those of state 1. On the other hand, the vigilance level of operator 2 has changed when the operator performed the most difficult task.

Moreover, the state probability of state 1 is increased as the difficulty level of a task increases. By performing correlation analysis, it is shown that there is a positive correlation between the difficulty level of a task and the vigilance level of an operator at the 5% significance level. The result corresponds to the research by [29], where the vigilance level and the task difficulty are shown to be positively correlated.

### 4.2. Model Evaluation

In this section, the performance of the proposed hypovigilance detection models is evaluated by using EEG signal obtained from subjects while performing tasks in Falcon 4.0 with two levels of difficulty.

#### 4.2.1. Dataset

For the evaluation, experiments with five subjects have been conducted. The detailed information on the subject participated and experiment environment is presented in Table 3. In order to make the experiments similar to the real-world situation, we have carefully chosen the five subjects. One of the subjects is a pilot and four of the subjects have much experience in operating flight simulators. They have also been trained before participating in the experiments.

Subjects performed tasks provided from Falcon 4.0 flight simulator by means of a joystick and a keyboard on a cockpit. Specifically, there were two joysticks for steering and two screens for displaying information relevant to the flight status. In addition to the visually transmitted information, subjects also received audio signal from the control tower through a headset.

For obtaining the EEG signal, a device called Mindset, one of the commercial EEG-signal acquisition devices for researchers and developers, is utilized. The device is equipped with four electrodes on its headset, where the electrodes are placed on the forehead of a subject. Note that we have altered EEG-signal acquisition device to provide comfortable experiment environment through utilizing smaller number of electrodes of Mindset compared to that of Emotiv EPOC. Nonetheless, the experiment results are the same since EEG signal acquired by both devices has been shown to have no significant difference [20]. Besides, even if there exist minor differences, it does not affect the performances of the proposed model as long as training and test data is generated from the same EEG-signal acquisition device. After collecting EEG signal, noises from the surface scalp of the subjects were removed by a noise removal function embedded in the device, and artifact rejection was conducted through visual inspection.

**Figure 7:**
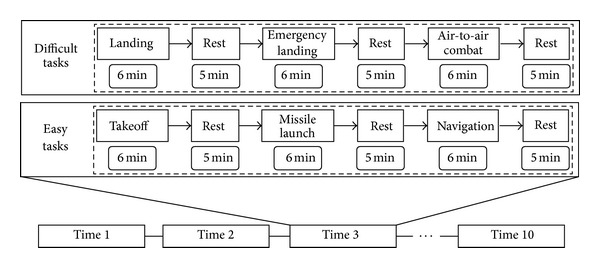
Data acquisition process which consists of tasks in two levels of difficulty, where each task takes about six.

According to the level of difficulty, six tasks, landing, emergency landing, air-to-air combat, taking off, missile launch, and navigation, were divided into two groups, resulting in landing, emergency landing, and air-to-air combat to be in the difficult task group and the rest of tasks in the easy task group. The detailed steps involved in each task are shown in Tables 4 and 5.  Figure 7 depicts the overall data acquisition process of the experiment. The five subjects repeatedly performed the procedure 10 times, and each time, two groups of tasks were performed, the difficult task group and easy task group. To prevent the effect of fatigue while maneuvering, there were five-minute breaks between consecutive tasks.

#### 4.2.2. Evaluation Measure

To evaluate the performance of the proposed models, accuracy is used as an evaluation criterion which is one of the widely used measures to assess the classification capability of a model. It computes the degree of correctly classified instances by a model based on a confusion matrix that shows the number of correct and incorrect predications compared to the actual levels.

In a confusion matrix for the vigilance level classification shown in Table 6, TP (true positive) refers to the number of correctly detected hypovigilance instances, whereas FP (false positive) indicates the number of falsely detected hypovigilance instances. Similarly, TN (true negative) and FN (false negative), respectively, means the number of correctly and incorrectly detected normal-vigilance instances. Based on the confusion matrix, accuracy is calculated as follows:

(5) $$\begin{array}{c} \text{Accuracy}=\frac{\text{TP}+\text{TN}}{\text{TP}+\text{FP}+\text{FN}+\text{TN}}. \end{array}$$

#### 4.2.3. Experiment Results

For comparison purposes, four types of models, DM1, DM2, DM3, and DM4, and additional “All” model were implemented. The models differ from each other in terms of training ratio, 50% and 67%, and the number of states in HMMs, two and three, as summarized in Table 7. In addition, “All” model differs from the four models since it is trained by using aggregated sequences of EEG signal generated from all operators, while others use signals from individual operators for training separately.

Specifically, hypovigilance detection models proposed in this paper utilized observed data for training to learn patterns of EEG signal indicating vigilance levels. Determining the ratio of the training data to the whole data, called the training ratio, is important as there is a tradeoff between performance improvement and overfitting problem. In the previous literatures, training ratios between 50% and 80% are widely used [64]. Therefore, in our research, we have chosen two types of training ratio, 50% and 67%, for comparison.

We have chosen two and three state HMMs for comparison since they are most widely used and successfully applied to diverse domains [65, 66]. The number of states in an HMM indicates hidden factors explaining vigilance level of the HMM. For instance, when an HMM referring to hypovigilance state is given, states in the HMM may imply causes for the hypovigilance such as fatigue or intrusion. While more sophisticated modeling of EEG-signal sequences is possible as the number of states in an HMM increases, an overfitting problem may occur. In case of a three-state HMM, three distribution functions are used for modeling of the given data, resulting in more precise modeling compared to a two-state model as long as it is addressed properly.

**Figure 8:**
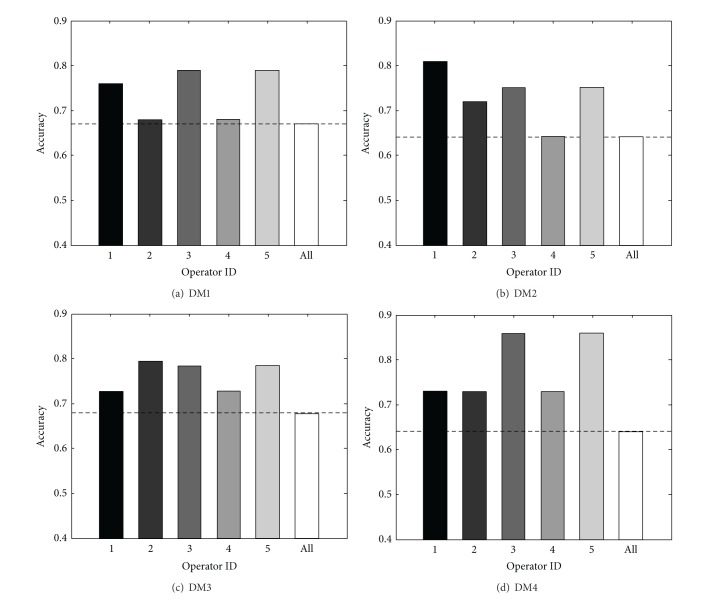
Accuracies of four models, DM1, DM2, DM3, and DM4, for five operators with “All” model.

The performance comparison results of the four models, DM1, DM2, DM3, and DM4, shown in Figure 8, suggest the following. First, hypovigilance detection models trained by using sequences of EEG signal from a single operator outperform a model trained by using aggregated sequences from diverse operators. The accuracy of “All” model depicted by a broken line and a white bar in graphs is always lower than accuracies of all the individual operators. Second, the suitability of adopting EEG signal based hypovigilance detection models varies among individuals. For instance, accuracies for operators 3 and 5 are always higher than those of other operators, and those for operator 4 are always the worst. Moreover, the best performing model among the four models that were compared differs depending on the operators. For operator 1, DM1 performed the best, while it showed the worst performance for operator 2; in contrast, DM3 was the best for operator 2.

Table 8 shows the accuracy improvements of four models over “All” model averaged across operators. It can be observed that models with the higher training ratios show better results. In addition, on average, models with three-state HMMs result in better performances over two-state HMMs such that the difference of improvement between DM1 and DM2 is about 1.5%, while that between DM3 and DM4 is more than 10%. From the results of the one-sided* t*-test, it can be concluded that accuracies of the proposed models are expected to be higher than 0.7 at the 5% significance level, implying that the proposed models in this paper show satisfactory performances.

## 5. Conclusions

Recently, the number of operators for UCAVs has increased with the development in military technology. The remote control of the aerial vehicles contributes to the elimination of human casualties and the expansion of operational ranges. However, high accident rate becomes a significant issue in deploying UCAVs. It has been revealed that one of the main reasons for high accident rate is a hypovigilance problem occurring when the level of vigilance declines, while an operator is carrying out operational tasks. Considering that an UCAV operator controls a vehicle in a remote cockpit, it can be difficult for an operator to sustain an appropriate level of vigilance over time. Therefore, detecting hypovigilance of an UCAV operator is important.

To address the above problem, we proposed HMM-based hypovigilance detection models for UCAV operators based on EEG signal. In the models, detection is done by means of HMMs which have capability of dealing with dynamics in sequential data. Different detection models for individual operators are trained, and, in each detection model, two HMMs representing hypovigilance and normal vigilance are utilized.

Experiments were conducted to measure the efficacy and the effectiveness of the proposed models. From the first set of experiments, the vigilance level hypothesis which indicates that higher vigilance is required for performing tasks with higher level of difficulty was validated, and the results from the second set of experiments demonstrated that the performance of the proposed model is satisfactory.

There are three contributions of the paper. First, to the best of the authors' knowledge, this research is the first model that tries to detect hypovigilance of UCAV operators, and we conducted an extensive survey on the previous literatures to find the most suitable method to address the problem. Second, we have suggested the possibility of using the difficulty levels of tasks as the vigilance levels of UCAV operators by adopting the vigilance level hypothesis to address the problem of absence of unanimously agreed criteria for vigilance levels. Last, we have validated the effectiveness and robustness of the proposed method by performing a systematic experimentation with realistic settings, experienced subjects, and commercial EEG-signal acquisition devices.

For future work, we intend to further develop more sophisticated models by conducting a large-scale experiment with more subjects and finding an optimal number of states for HMMs to enhance the performance of hypovigilance detection. Moreover, professional operators tend to be more familiar with specific tasks than inexperienced persons, requiring different criteria for measuring the difficulty levels for the tasks. As such, when professional operators are involved in conducting the tasks, it is necessary to consider how to determine the different difficulty levels for tasks for them. To obtain more precise experiment results from different subjects, appropriate difficult levels would need to be defined before conducting the experiments. The proposed hypovigilance detection models can contribute to lowering the accident costs for operations of UCAVs.

## Figures and Tables

**Algorithm 1:**
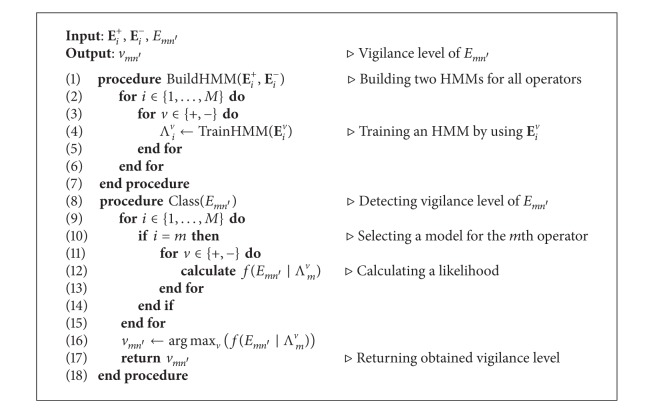
Pseudocode for detecting the vigilance level of a given EEG-signal sequence, *E*_*mn*′_, of an operator, *o*_*m*_, performing a task, *t*_*n*′_.

**Table 1:** Previously proposed hypovigilance detection methods categorized based on the data type used in studies along with their characteristics.

Data type	Data example	Characteristic	Reference
Survey data	Karolinska sleepiness scale (KSS)	Easy to obtain and understand; existence of possible bias; intrusive	[34, 35]
Physical data	Eye blink duration; eye blink amplitude; amplitude and velocity ratio of eye blink	Possible to be real time; less intrusive; existence of possible bias	[30, 36]
Performance data	Road center position; steering wheel angle; vehicle velocity	Possible to be real time; less intrusive; existence of possible bias	[31, 37]
Biological data	Electroencephalography (EEG); electrocardiogram (ECG); electrooculography (EOG)	Possible to be real time; less intrusive; less bias; sensitive to environment	[33, 39, 40]

**Table 2:** Previous studies on EEG-signal classification categorized into static and dynamic classifiers presented with their methods and applications.

Category	Reference	Classification method	Application
Static	[46]	Support vector machines; neural networks	Emotion detection
Static	[48]	Naïve Bayes; Fisher discriminant analysis	Emotion detection
Static	[44]	Support vector machines	Driver drowsiness detection
Static	[17]	Support vector machines; Gaussian mixture model; neural network	Epileptic activity detection
Dynamic	[50]	HMMs	Driver drowsiness detection
Dynamic	[49]	Time-delay neural network; gamma neural network	EEG-signal change detection
Dynamic	[51]	HMM; input-output HMM	Mental task classification

**Table 3:** Description of five subjects participated in the experiments and experiment environment.

	Age	Sleep hours (hrs)	Recent disorder	Temperature (°C) margin of error ± 1
1	30	6	Headache	26.5
2	31	7	None	26.5
3	28	5	None	26
4	26	8	None	25.5
5	28	8	None	25.5

**Table 4:** Subordinate steps of tasks with a high level of difficulty, landing, emergency landing, and air-to-air combat.

Step	Landing	Emergency landing	Air-to-air combat
1	Request permission to land	Warn reset	Switch to 2D cockpit view
2	Check assigned runway	Throw abandoned armaments	Select air-to-air radar mode
3	Check current speed/pitch	Check current speed/pitch	Keep speed/altitude/direction
4	Check current altitude	Keep speed/pitch	Check target on the radar
5	Landing gear down	Declare emergency landing	Target lock on
6	Decelerate	Landing gear down	Launch
7	Keep speed/pitch	Speed brake open	Check speed/altitude/direction
8	Flare	Check the tires touch down	Search target
9	Check the tires touch down	Hold pitch attitude	Flare

**Table 5:** Subordinate steps of tasks with a low level of difficulty, taking off, missile launch, and navigation.

Step	Takeoff	Missile launch	Navigation
1	Check permission to takeoff	Switch A-G mode	Set up steer point/time
2	Set throttle to max	Masters arm on	Set up speed/altitude/direction
3	Check current speed	Select armaments	Keep speed/altitude/direction
4	Pitch up	Missile on	Check remaining distance
5	Check speed	Remove lens cover	Check arrival at steer point
6	Landing gear up	Seeker on	Set up other steer points/time
7	Check radar	Check launch zone indicator	Turn
8	Enter orbit	Target lock on	
9		Check speed/altitude	

**Table 6:** Confusion matrix for vigilance level classification.

	Predicted positive	Predicted negative
Actual positive	True positive (TP)	False negative (FN)
Actual negative	False positive (FP)	True negative (TN)

**Table 7:** Summary of the four models, DM1, DM2, DM3, and DM4, in terms of the number of states in HMMs and training ratio.

Classifier	The number of states in HMMs	Training ratio (%)
DM1	2	50
DM2	2	67
DM3	3	50
DM4	3	67

**Table 8:** Average improvements of the four models, DM1, DM2, DM3, and DM4, over “All” model for each operator in terms of accuracy.

Classifier	Average improvement (%)
DM1	10.45
DM2	11.88
DM3	12.94
DM4	22.19
